# Automated deep-learning system in the assessment of MRI-visible prostate cancer: comparison of advanced zoomed diffusion-weighted imaging and conventional technique

**DOI:** 10.1186/s40644-023-00527-0

**Published:** 2023-01-17

**Authors:** Lei Hu, Caixia Fu, Xinyang Song, Robert Grimm, Heinrich von Busch, Thomas Benkert, Ali Kamen, Bin Lou, Henkjan Huisman, Angela Tong, Tobias Penzkofer, Moon Hyung Choi, Ivan Shabunin, David Winkel, Pengyi Xing, Dieter Szolar, Fergus Coakley, Steven Shea, Edyta Szurowska, Jing-yi Guo, Liang Li, Yue-hua Li, Jun-gong Zhao

**Affiliations:** 1 Department of Diagnostic and Interventional Radiology, Shanghai Sixth People’s Hospital Affiliated to Shanghai Jiao Tong University School of Medicine, No. 600, Yi Shan Road, Shanghai, 200233 China; 2 MR Application Development, Siemens Shenzhen magnetic Resonance Ltd., Shenzhen, China; 3 Department of Radiology, Xiangyang No.1 People’s Hospital, Hubei University of Medicine, Xiangyang, 441000 China; 4 MR Application Predevelopment, Siemens Healthcare GmbH, Erlangen, Germany; 5 Innovation Owner Artificial Intelligence for Oncology, Siemens Healthcare GmbH, Erlangen, Germany; 6 Digital Technology and Innovation, Siemens Healthineers, Princeton, NJ USA; 7 Radboud University Medical Center, Nijmegen, Netherlands; 8 New York University, New York City, NY USA; 9 Charité, Universitätsmedizin Berlin, Berlin, Germany; 10 Eunpyeong St. Mary’s Hospital, Catholic University of Korea, Seoul, Republic of Korea; 11 Patero Clinic, Moscow, Russia; 12 Universitätsspital Basel, Basel, Switzerland; 13 Changhai Hospital, Shanghai, China; 14 Diagnostikum Graz Süd-West, Graz, Austria; 15 Oregon Health and Science University, Portland, OR USA; 16 Loyola University Medical Center, Maywood, IL USA; 17 Medical University of Gdansk, Gdansk, Poland; 18 Clinical Research Center, Shanghai Sixth People’s Hospital Affiliated to Shanghai Jiao Tong University School of Medicine, Shanghai, 200233 China; 19 Department of Radiology, Renmin Hospital of Wuhan University, Wuhan, 430060 China

**Keywords:** Diffusion magnetic resonance imaging, Deep learning, Prostatic neoplasms, Artifact, Risk factor

## Abstract

**Background:**

Deep-learning-based computer-aided diagnosis (DL-CAD) systems using MRI for prostate cancer (PCa) detection have demonstrated good performance. Nevertheless, DL-CAD systems are vulnerable to high heterogeneities in DWI, which can interfere with DL-CAD assessments and impair performance. This study aims to compare PCa detection of DL-CAD between zoomed-field-of-view echo-planar DWI (z-DWI) and full-field-of-view DWI (f-DWI) and find the risk factors affecting DL-CAD diagnostic efficiency.

**Methods:**

This retrospective study enrolled 354 consecutive participants who underwent MRI including T2WI, f-DWI, and z-DWI because of clinically suspected PCa. A DL-CAD was used to compare the performance of f-DWI and z-DWI both on a patient level and lesion level. We used the area under the curve (AUC) of receiver operating characteristics analysis and alternative free-response receiver operating characteristics analysis to compare the performances of DL-CAD using f- DWI and z-DWI. The risk factors affecting the DL-CAD were analyzed using logistic regression analyses. *P* values less than 0.05 were considered statistically significant.

**Results:**

DL-CAD with z-DWI had a significantly better overall accuracy than that with f-DWI both on patient level and lesion level (AUC_patient_: 0.89 vs. 0.86; AUC_lesion_: 0.86 vs. 0.76; *P* < .001). The contrast-to-noise ratio (CNR) of lesions in DWI was an independent risk factor of false positives (odds ratio [OR] = 1.12; *P* < .001). Rectal susceptibility artifacts, lesion diameter, and apparent diffusion coefficients (ADC) were independent risk factors of both false positives (OR_rectal susceptibility artifact_ = 5.46; OR_diameter,_ = 1.12; OR_ADC_ = 0.998; all *P* < .001) and false negatives (OR_rectal susceptibility artifact_ = 3.31; OR_diameter_ = 0.82; OR_ADC_ = 1.007; all *P* ≤ .03) of DL-CAD.

**Conclusions:**

Z-DWI has potential to improve the detection performance of a prostate MRI based DL-CAD.

**Trial registration:**

ChiCTR, NO. ChiCTR2100041834. Registered 7 January 2021.

## Background

Diffusion-weighted imaging (DWI) is an indispensable technique in prostate magnetic resonance imaging (MRI), providing both qualitative and quantitative functional information of prostate tissue and lesions [1–5]. Relying on the Prostate Imaging Reporting and Data System (PI-RADS) [2], DWI combined with T2-weighted imaging (T2WI) has shown significantly improved accuracy of detection and characterization of prostate cancer (PCa) lesions [6] and plays an important role in the clinical management strategy of patients with suspected PCa. However, due to differences in hardware, software, and technical experience [7, 8], there is a high variation of diagnostic accuracy and inter-observer agreement in the interpretation of prostate MRI across medical centers [1, 2]. These factors limit the clinic application of prostate MRI.

Various deep-learning-based computer-aided diagnosis (DL-CAD) systems using prostate MRI for PCa detection have demonstrated comparable or improved performance and reproducibility with less time and labor compared to experienced radiologists [7, 9–12]. Nevertheless, DL-CAD systems are vulnerable to high heterogeneities in DWI [13], which can interfere with DL-CAD assessments and impair performance. Developing a DWI sequence that can improve the accuracy and reliability of DL-CAD would improve PCa diagnosis. DWI acquisition performed using full-field-of-view (FOV) ssEPI-DWI (f-DWI) is prone to distortions, susceptibility artifacts, and limited spatial resolution. By contrast, zoomed-field-of-view echo-planar DWI (z-DWI) using a small FOV that only covers a specific region-of-interest (ROI) results in fewer geometric distortions, susceptibility artifacts, and higher spatial resolution [2, 10, 14–18]. Previous studies indicated that variation of noise, deformation and changes of resolution are important factors interfering with the judgement of DL-CAD [13, 19–22], therefore, we hypothesized that z-DWI might be helpful to improve the performance of DL-CAD for PCa diagnosis.

In this study, we assessed the use of DL-CAD with f-DWI and with z-DWI and compared each format in diagnosing MRI-visible PCa. The risk factors of patient condition, image quality, and lesion characteristics that could affect the DL-CAD diagnostic efficiency were analyzed.

## Methods

This retrospective study was approved by the local ethics committee at our institution [Approve No: 2022-KY-073(K)]. As part of a prospective study aiming to build a robust AI system for PCa diagnosis, all the enrolled subjects signed the informed consent before they underwent the MRI examination and allowed us to use their data for a series of follow-up studies about AI system building for PCa diagnosis. All procedures performed in studies involving human participants were according to the 1964 Helsinki Declaration and its later amendments.

### Participant selection

Between January 2021 and January 2022, participants with clinically suspected PCa undergone MRI examinations and subsequent MRI fusion ultrasound-guided targeted biopsies (2–4 cores) of MRI-suspicious lesions (PI-RADS score ≥ 3) followed by systematic biopsies (10–12 cores) were consequently enrolled from Shanghai Sixth People’s Hospital Affiliated to Shanghai Jiao Tong University School of Medicine. All MRI scans were interpreted by two senior radiologists with more than 15 years of experience in prostate MRI interpretation using the PI-RADS version 2.1.

The inclusion criteria included the following: (a) prostate lesions with definite boundaries on three types of MR images according to according to PI-RADS version 2.1 (T2WI, DWI, and ADC); (b) complete clinical information and entire MRI reports including the number, PI-RADS score, and location of suspected PCa lesion; (c) complete biopsy records and results, including the number, location, Gleason score (GS) of lesions. Exclusion criteria were (a) a prior history of PCa treatment; (b) biopsy within 6 months prior to the MRI examination; (c) an interval of more than 2 weeks between MRI and the biopsy procedure; (d) unavailability of the final PCa diagnosis.

### MRI examination

Patients were advised to empty their bowel prior to the examination. All patients underwent both T2-weighted imaging (T2WI), f-DWI, z-DWI with b-values of 50, 1000, and 1500 s/mm^2^ on a 3 T MRI scanner (MAGNETOM Skyra, Siemens Healthcare, Erlangen, Germany), and a phased-array 18-channel body coil in combination with an integrated 32-channel spine coil was used for signal reception. Z-DWI was performed with a slight rotation of the field-of-excitation [16], motion registration [23], and complex averaging [24]. More detailed parameters of each MRI sequence are shown in Table 1.

**Table 1:** MR sequence parameters

Parameter	T2-weighted imaging	f-DWI	z-DWI
Sequence	TSE	SS-EPI	zoomed SS-EPI
Field of view (mm^2^)	200 × 200	380 × 280	190 × 109
Scan matrix	320 × 256	178 × 132	100 × 56
Voxel size (mm^3^)	0.6 × 0.6 × 3.5	2.1 × 2.1 × 3.0	1.9 × 1.9 × 3.0
PE Dir.	Left- > Right	Anterior- > Posterior	Left- > Right
Echo time (ms)	101	79	76
Repetition time (ms)	6000	4200	3800
Bandwidth (Hz/px)	200	1872	1612
b-value (s/mm^2^)	NA	50, 1000, 1500	
NEX	2	1, 3, 6	
Fat suppression	NA	FatSat	
Acceleration factor	2 (GRAPPA)	2 (GRAPPA)	NA
Acquisition time (min)	2:08	3:05	2:37

*PE Dir.* Phase Encoding direction, *GRAPPA* Generalized auto-calibrating partial parallel acquisition, *NA* not applicable

### Histopathology matching and annotation

The ground truth of this study was lesion confirmation on histopathology after biopsy. At least one GU radiologist and one GU pathologist retrospective reviewed MRI and histopathology examinations together at a multidisciplinary meeting scheduled monthly. Each lesion in MRI was matched to the corresponding location on the specimen through visual co-registration. According to the Ginsburg Study group method, a sextant scheme of the prostate has been used for analysis of the correct identification of the lesions’ localization [25]. Prostate contours were segmented on T2WI images and partitioned into sextants using the midsagittal plane and four additional angulated planes according to the biopsy protocol. Sextant-specific systematic biopsy histopathology was assigned to all MR sextants and augmented by calculating the maximum GS between systematic histopathology and histopathology from targeted fusion biopsy to sextants intersecting with MR lesions to create a sextant map of histopathology ground truth [26]. MRI reports and biopsy results, including the number, GS, and prostate location by MRI of lesions of all selected participants, were recorded in the regular retrospective review.

### Deep learning-based computer-aided diagnosis

A prototype DL-CAD system (MR Prostate AI v1.2.5; October 2020; Siemens Healthcare GmbH, Erlangen, Germany) was used to test the performances of z-DWI and f-DWI in PCa detection. This DL-CAD system was trained using 2170 bp-MRI prostate examinations from 7 institutions, consisting of 944 lesion-free cases and 1226 cases with at least 1 clinically significant lesion that is deemed at least equivocal as designated by PI-RADS score 3 and higher; none of the cases included in the testing data set was part of the training [9]. The architecture and processing steps of the DL-CAD system have been described in detail in previous studies [9, 12, 27]. In brief, the system computes apparent diffusion coefficient (ADC) maps and calculates b-value images at b = 2000 s/mm^2^ using the input DWI and then segments whole-gland volumes using T2WI. After that, the DWI and ADC are aligned to the T2WI. Finally, the system identifies the clinically relevant lesions based on T2WI, calculated b-value images, ADC maps, and prostate segmentations, and provides detected lesion localization, a PI-RADS category, and case-based level of suspicion (LoS). The LoS represents the confidence of the software that a lesion with a PI-RADS score of 3 or above is present in that patient, and ranges from 3.0 to 5.0 in steps of 0.1.

### Evaluation of detection performance

The patient-based diagnostic performance of DL-CAD was evaluated by computing ROC curves for each case-based LoS. We evaluated two clinically relevant tasks: (a) differentiating between benign lesion and PCa (Gleason Grade Group (GGG) ≥ 1), and (b) differentiating between benign lesion or low-grade cancer of GS 3 + 3 and clinically significant PCa (csPCa) (GGG ≥ 2) [11].

The DL-CAD system can automatically detect clinically relevant PCa lesions which were defined on pathology/histology as Gleason score ≥ 7, and/or volume ≥ 0.5 cc, and/or extra-prostatic extension according to PI-RADS v2.1. The clinically relevant PCa lesions-based detection performances of DL-CAD were evaluated using free-response receiver operating characteristics (FROC) analysis due to PCa’s multi-focality [6, 28, 29]. In addition, considering the FROC curve has an infinite area under the curve (AUC), we also used alternative free-response receiver operating characteristics (AFROC) analysis with a finite AUC ranging from 0 to 1 to evaluate the lesion-based detection performance of the DL-CAD [30].

### DWI image analysis

Two radiologists with approximately 3 years of experience in prostate MRI reporting and blinded to all clinical details and biopsy results twice evaluated the DWI sets including the image quality scoring, DWI signal intensities measurements and ADC measurements. The independent readings of the first time were used for assessing inter-reader agreement on quality scores, and DWI signal intensities and ADC measurements. The final image quality score of each DWI set was determined by the two radiologists by consensus. The final image quality score and the mean results of DWI signal intensities measurements and ADC measurements of the two radiologists were used for the evaluation of risk factors affecting the DL-CAD diagnostic efficiency.

After four weeks, the second time image analysis was performed by the two readers in a different order to test for reproducibility. Image evaluation was performed using the Image J Software (National Institutes of Health, Bethesda, MD, USA).

For each time, the DWI sets in two different sessions at intervals of at least two weeks to minimize recognition bias. In each session, only 1 of the 2 DWI sets for each patient was evaluated. Specifically, the DWI sets were reviewed in a random order and were rated in terms of overall quality and anatomic distortion using a 5-point Likert scale with 5 indicating the highest quality [18, 31]. Axial T2WI images were used as a reference for guiding anatomical localization of findings on the DWI sets [32]. In addition, the presence of artifacts including rectal susceptibility artifacts, phase wrap-around, artifacts from artificial joint replacements, other artifacts from outside the body (only for f-DWI) was noted, and the grade of artifact influence on image quality was scored as: 1, excellent image quality; 2, mild artifact, not impacting diagnosis; 3, moderate artifact, mildly impacting diagnosis; 4, pronounced artifact, moderately impacting diagnosis; 5, pronounced artifact, non-diagnostic. Artifacts scored ≥3 were considered to have an influence on the diagnosis [10].

To evaluate noise, lesion conspicuity, and ADC values of each DWI set, the radiologists were also asked to draw ROIs on the ADC map, which were then copied to the DWI (b = 1500 s/mm^2^) image. One ROI was placed in the center of the lesion in the slice with the largest extent of the lesion. The second ROI was placed in the corresponding contralateral normal tissue as a reference ROI. If the contralateral tissue was also abnormal, the reference ROI was placed in healthy appearing tissue of the same anatomical zone as the lesion. To calculate the standard deviation of the noise (SD_noise_) in a noise-only area, a third ROI was placed in the center of the bladder with the DWI at b = 1500 s/mm^2^. The ADC values, mean signal intensities in the lesion ROI (S_lesion_), the reference ROI (S_normal_), and SD_noise_ were recorded for further analyses.

The difference in the noise between z-DWI and f-DWI was calculated using the estimated signal-to-noise ratio (eSNR) [33]:

$$eSNR={S}_{lesion}/S{D}_{noise}$$

Lesion conspicuity was determined by the contrast-to-noise ratio (CNR):

$$CNR=\left({S}_{lesion}-{S}_{normal}\right)/S{D}_{noise}$$

We compared DWI quality and the characteristics of benign lesions and malignant lesions to determine risk factors affecting the DL-CAD diagnostic efficiency. The relationships between DL-CAD diagnostic performance and image quality as well as lesion characteristics were also evaluated.

### Statistical analyses

The one-sample *Kolmogorov-Smirnov* test was used to check the assumption of a normal distribution of the data. The independent *t* test or paired *t* test was used for normally distributed data. The Mann-Whitney *U* test was used to assess non-normally distributed continuous variables. Categorical variables were reported as percentages and compared by χ^2^ test. Comparisons of sensitivity and specificity were performed using the McNemar test. Comparisons of AUCs were performed using the Delong test. Inter- and intra-observer agreement of overall quality, anatomic distortion, and artifact evaluation were tested with a weighted κ coefficient. ADC values and SI_lesion_ and SI_normal_ measurements were assessed with the intraclass correlation coefficient.

Univariable logistic regression analyses and multivariable logistic regression analyses with stepwise approaches were applied to assess the relationship between false positives and potential risk factors and between false negatives and potential risk factors. The multicollinearity of variables in the multivariable analysis was determined using a variance inflation factor (VIF) of greater than 10.

Statistical evaluations were performed using R v4.10 (R Foundation for Statistical Computing, Vienna, Austria; https://www.R-project.org/). The VIFs were calculated using the “car” package. Comparison of Binary Diagnostic Tests in a Paired Study Design was performed using “DTComPair” package. The ROC curves were plotted using the “pROC” package. The FROC curves and AFROC curves were plotted using the “BayesianFROC” package. Forest plots of the logistic regression analyses were performed using “forestmodel” package. *P* values less than 0.05 were considered statistically significant.

## Results

### Participant and lesion baseline characteristics

**Fig. 1:**
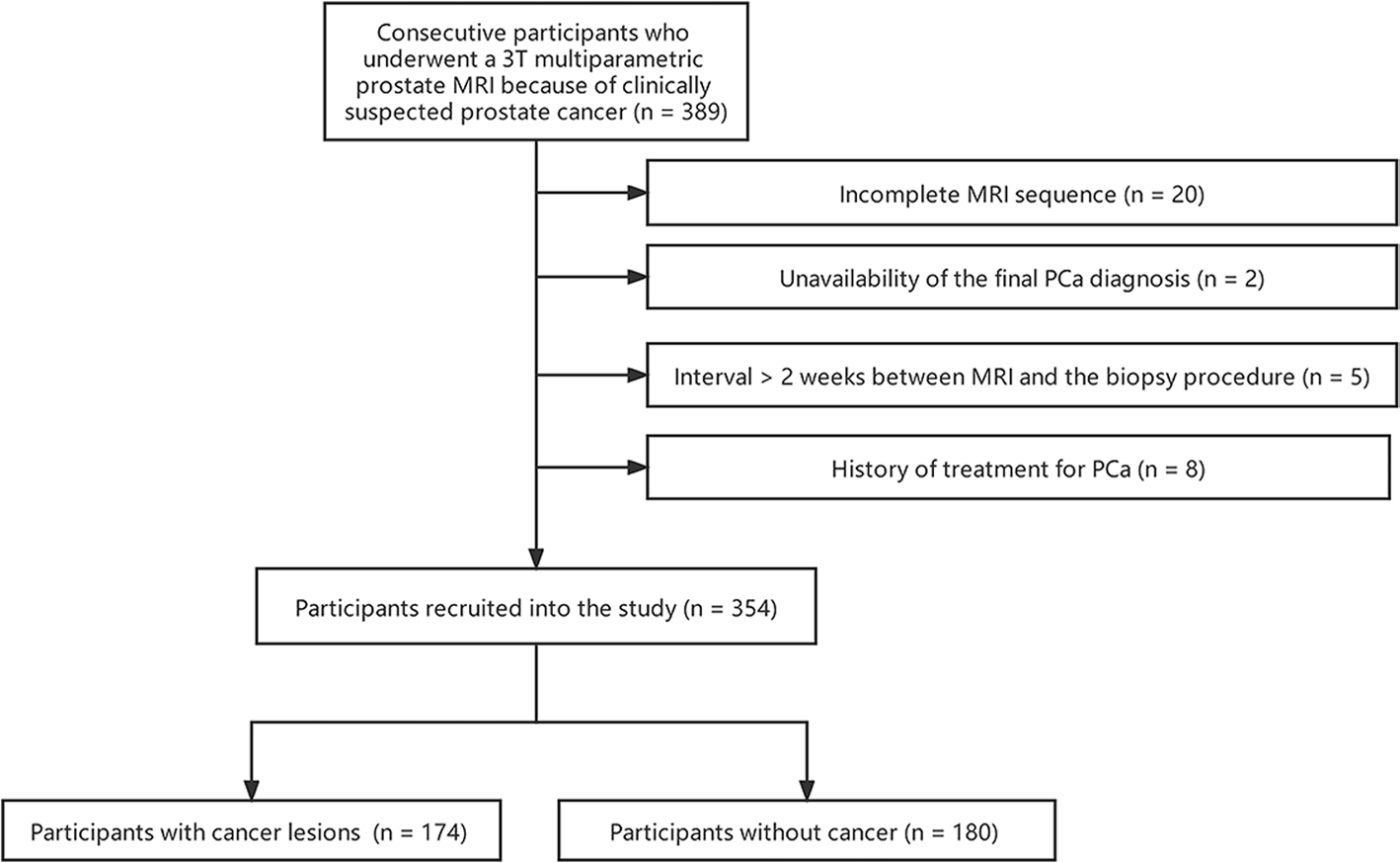
Flowchart of participant inclusion and exclusion

Initially, a total of 389 participants were enrolled. Of these, 35 were excluded according to the inclusion and exclusion criteria. The detailed reasons for exclusion are listed in Fig. 1. A total of 354 patients (median age, 65 years; interquartile range [IQR], 71–77 years) with 486 lesions (250 cancer lesions and 236 benign lesions) were included in the final study. Baseline epidemiologic and clinical characteristics of the participants are shown in Table 2.

**Table 2:** Demographic and Clinical Characteristics of Included Patients

Variable	Patients without cancer (*n* = 180)	Patients with cancer (*n* = 174)
Median age (year)^a^	69 (65 - 75)	72 (66 - 79)
Total PSA (ng/ml)^a^	8.9 (5.1 - 13.0)	14.0 (6.7 - 29.6)
Free PSA (ng/ml)^a^	1.3 (0.8 - 2.3)	1.6 (0.8 - 3.5)
Free PSA/total PSA^a^	0.2 (0.1 - 0.2)	0.1 (0.1 - 0.2)
No. of patients with MRI-detected lesions^b^		
1 lesion	145 (81)	107 (61)
2 lesions	21 (12)	59 (34)
3 lesions	8 (4)	7 (4)
More than 3 lesions	6 (3)	1 (1)

*PSA* prostate-specific antigen, *PI-RADS* Prostate Imaging Reporting and Data System

^a^Data in parentheses are the interquartile range

^b^Data in parentheses are percentages

There were no significant differences in the mean patient age between subjects with and without PCa (*P* = 0.23). Participants with PCa had higher levels of total PSA and free PSA and lower free PSA ratio than those without PCa (All *P* < .001). Detailed information about lesions, including lesion location, pathologic findings, and clinical assessment, is shown in Table 3.

**Table 3:** Lesion characteristics

Variable	Benign lesions (*n* = 236)	Malignant lesions (*n* = 250)
Lesion location^b^		
Transition zone	73 (31)	118 (47)
Peripheral zone	163 (69)	132 (53)
Median Diameter (mm)^a^	7.5 (4.6–11.8)	21.3 (13.1–37.3)
Clinical PI-RADS score†		
PI-RADS 2	123 (52)	23 (9)
PI-RADS 3	52 (22)	25 (10)
PI-RADS 4	47 (20)	110 (44)
PI-RADS 5	14 (6)	92 (37)
Gleason Group group†		
Gleason grade group 1 (GS 3 + 3)	NA	17 (7)
Gleason grade group 2 (GS 3 + 4)	NA	37 (15)
Gleason grade group 3 (GS 4 + 3)	NA	80 (32)
Gleason grade group 4 (GS = 8)	NA	33 (13)
Gleason grade group 5 (GS > 8)	NA	83 (33)

^a^Data in parentheses are the interquartile range

^b^Data in parentheses are percentages

### Patient-based performance

**Fig. 2:**
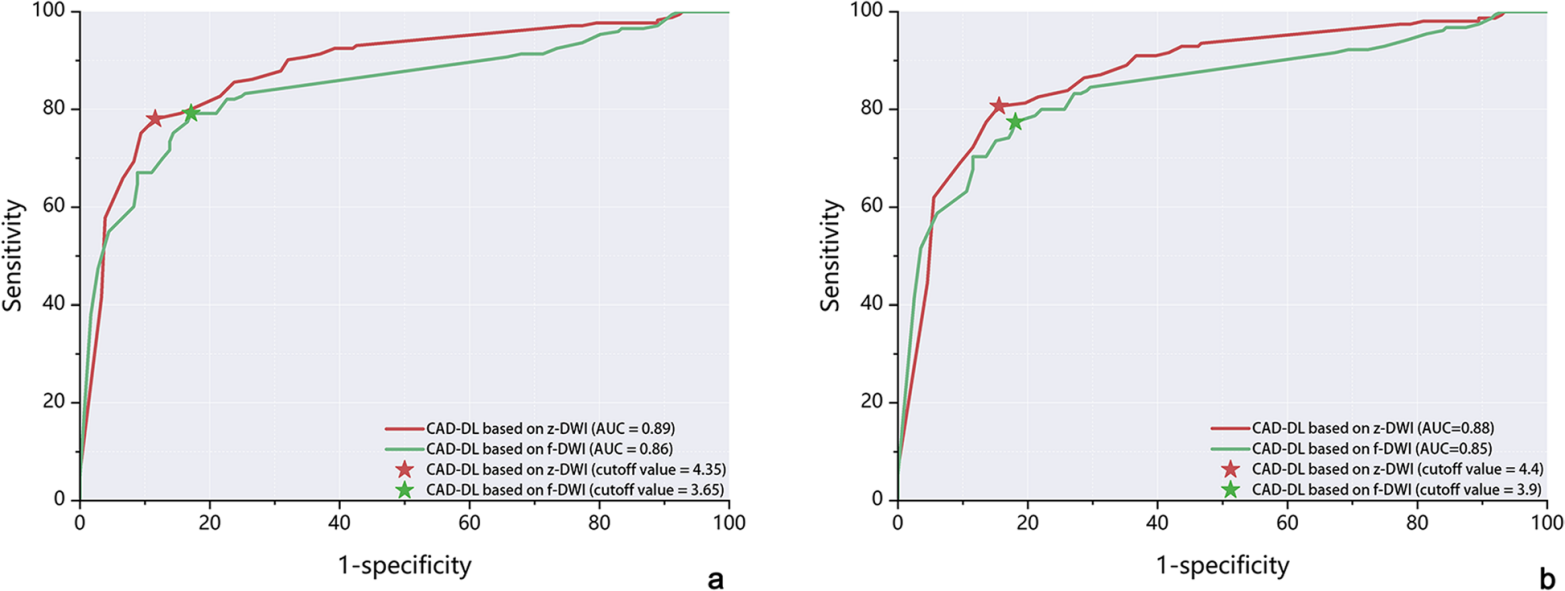
Comparison of receiver operating characteristics curves for analysis for LoS of DL-CAD based on f-DWI and z-DWI performance on benign versus grade group 1 and above (**a**) and on benign and grade group 1 versus grade group 2 and above (**b**). LoS = patient-based level of suspicion. DL-CAD = Deep Learning Based Computer-Aided Diagnosis

Figure 2 shows that, compared with f-DWI, DL-CAD based on z-DWI had better performance in differentiating between benign lesion and PCa (Sensitivity: 0.79 [95% CI: 0.73-0.85] vs. 0.78 [95% CI: 0.71-0.84]; Specificity: 0.89 [95% CI: 0.83-0.93] vs. 0.83[95% CI: 0.77-0.89]; AUC: 0.89 [95% CI:0.85-0.92] vs. 0.86 [95% CI: 0.81-0.89], *P* = 0.007) and in differentiating between benign tissue or PCa of GS 3 + 3 and csPCa (Sensitivity: 0.81 [95% CI: 0.74 - 0.87] vs. 0.78 [95% CI: 0.70-0.84]; Specificity: 0.85 [95% CI: 0.79-0.80] vs. 0.82 [95% CI:0.76-0.87]; AUC: 0.88 [95% CI:0.84-0.91] vs. 0.85 [95% CI: 0.81-0.88], *P* = 0.024).

### Lesion-based detection performance

**Fig. 3:**
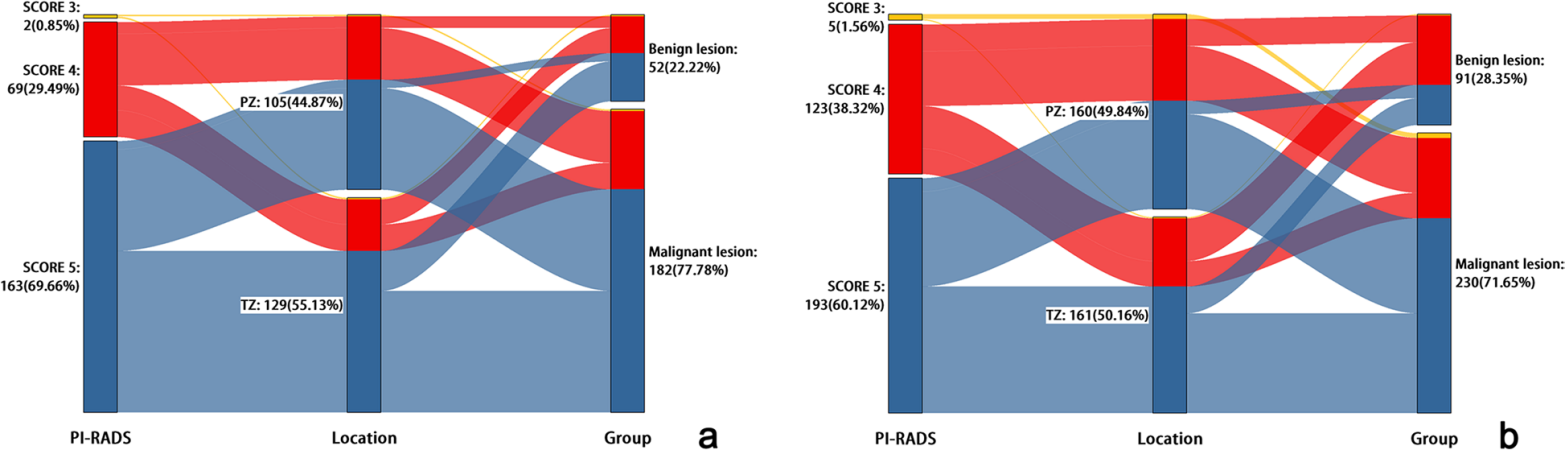
Alluvial diagrams showed all lesions detected and given PI-RADS score 3 (Yellow), PI-RADS score 4 (Red) and PI-RADS score 5 (Blue) by DL-CAD using f-DWI (**a**) and z-DWI (**b**). The detected lesions included both true-positive detections and false-positive detections. Compared with f-DWI, z-DWI has both more true-positive detections (182 vs. 230) and more false-positive detections (52 vs. 91)

Lesion-based detection performance of the DL-CAD system is shown in Table 4 and Fig. 3.

**Table 4:** Prostate cancer lesion detection performance of DL-CAD using f-DWI and z-DWI

PI-RADS	No. of true-positive detections		No. of false-positive detections	
	f-DWI (*n* = 182)	z-DWI (*n* = 230)	f-DWI (*n* = 52)	z-DWI (*n* = 91)
3	1	4	1	1
4	47	66	22	57
5	134	160	29	33

Compared with f-DWI, z-DWI had significantly higher sensitivity but lower specificity for lesion detection at PI-RADS category greater than or equal to 3 (Sensitivity: 0.93 [95% CI: 0.90-0.96] vs. 0.73 [95% CI:0.68-0.79]; Specificity: 0.61 [95% CI: 0.55-0.67] vs. 0.76 [95% CI:0.70-0.81]; *P* < .001 for all comparisons).

**Fig. 4:**
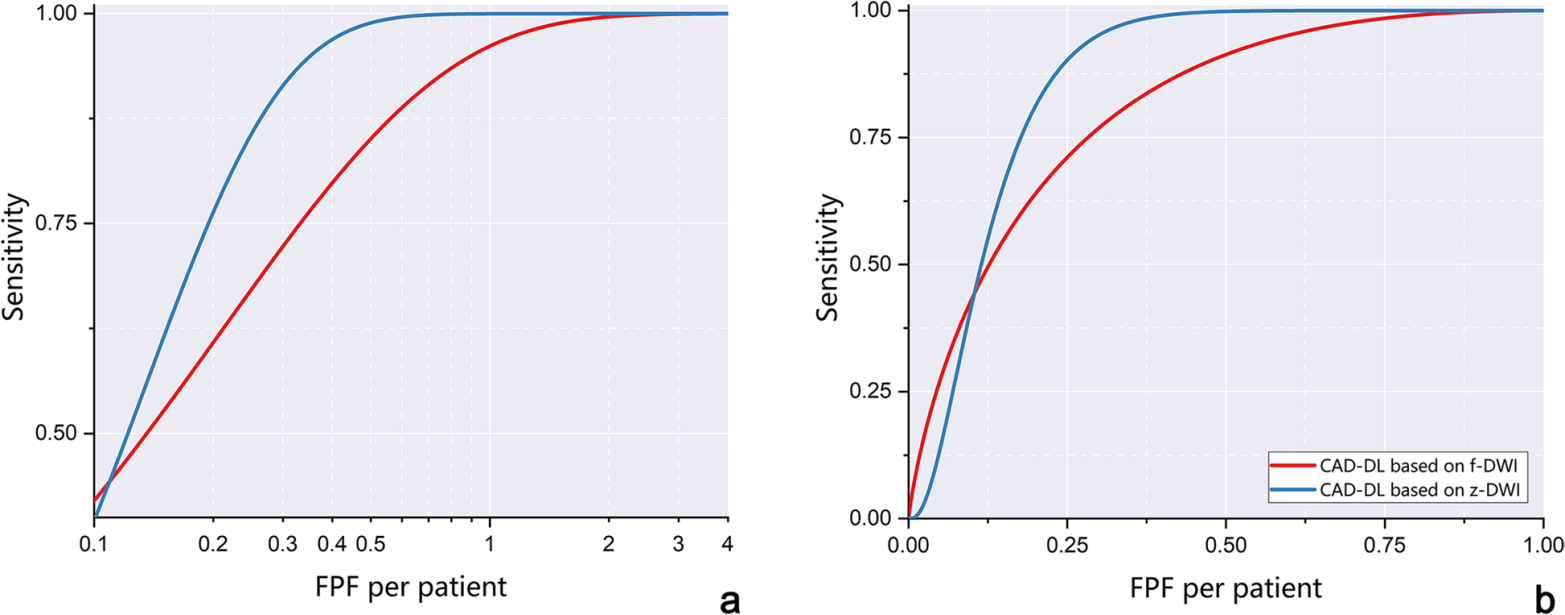
FROC analysis (**a**) and AFROC analysis (**b**) for detection sensitivity on prostate cancer lesions. The number of false positives (x-axis) of Fig. 4a is shown in log-scale. According to the FROC curves, for a detection sensitivity > 0.1, using z-DWI shows a lower rate of false-positive detections per patient. The AFROC curves (**b**) show that compared with using f-DWI, using z-DWI can result in higher AUC in terms of PCa lesion detections per patient (0.855 [95%CI:0.825-0.883] vs. 0.760 [95%CI:0.714-0.799]). FROC=Free-response receiver operating characteristics analysis. AFROC = alternative free-response receiver operating characteristics. FPF = false positive fraction

At a detection sensitivity > 0.1, DL-CAD using z-DWI provided lower False Positive Fractions per patient than DL-CAD using f-DWI (Fig. 4a). DL-CAD using z-DWI had better performance for PCa lesion detection with less false-positive detections per patient than DL-CAD f-DWI (AUC, 0.855 [95% CI:0.825-0.883] vs. 0.760 [95% CI: 0.714-0.799]; *P* < .001) (Fig. 4b).

### DWI image analysis

The inter- and intra-observer agreement for image quality scores, DWI signal intensities, and ADC measurements were concordant (Suppl. material).

As shown in Table 5, DL-CAD using z-DWI has significantly higher scores for overall image quality and distortion of the prostate, and lower scores of the severity of artifacts compared with DL-CAD using f-DWI (*P* ≤ .035). For both benign lesions and malignant lesions, DL-CAD using z-DWI had lower ADC values and higher CNR and eSNR than those in DL-CAD f-DWI (*P* ≤ .011).

**Table 5:** Comparison of subjective image quality and the main lesion between different diffusion-weighted imaging sequences of the prostate

Feature	f-DWI	z-DWI	*P* value
Subjective image quality score (*n* = 354)			
Overall image quality^b^	4.0 ± 0.8	4.4 ± 0.7	<.001
Distortion of prostate^b^	4.1 ± 0.7	4.5 ± 0.7	<.001
Rectal susceptibility artifact^b^	1.6 ± 0.9	1.5 ± 0.8	0.035
Artificial joint replacements^b^	1.1 ± 0.6	1.0 ± 0.2	<.001
Phase wrap-around^b^	1.3 ± 0.7	1.0 ± 0.1	<.001
Other artifacts out of body^b^	1.2 ± 0.6	NA	NA
Main lesion characteristics			
Benign lesion (*n* = 236)			
eSNR^a^	21.9 (17.5-29.2)	30.7 (25.1-40.4)	<.001
CNR^a^	1.6(1.0-3.6)	2.1(1.0-4.6)	0.011
ADC^b^	1027.6 ± 262.5	977.9 ± 272.7	<.001
Malignant lesion (*n* = 250)			
eSNR^a^	27.7(22.3-37.6)	44.2(33.1-57.7)	0.233
CNR^a^	7.6 (2.5-15.6)	8.0 (4.0-16.7)	0.004
ADC^b^	751.5 ± 205.3	698.3 ± 158.8	<.001

^a^Data in parentheses are the interquartile range. ^b^Numbers are means ± standard deviations

### Risk factors evaluation

**Fig. 5:**
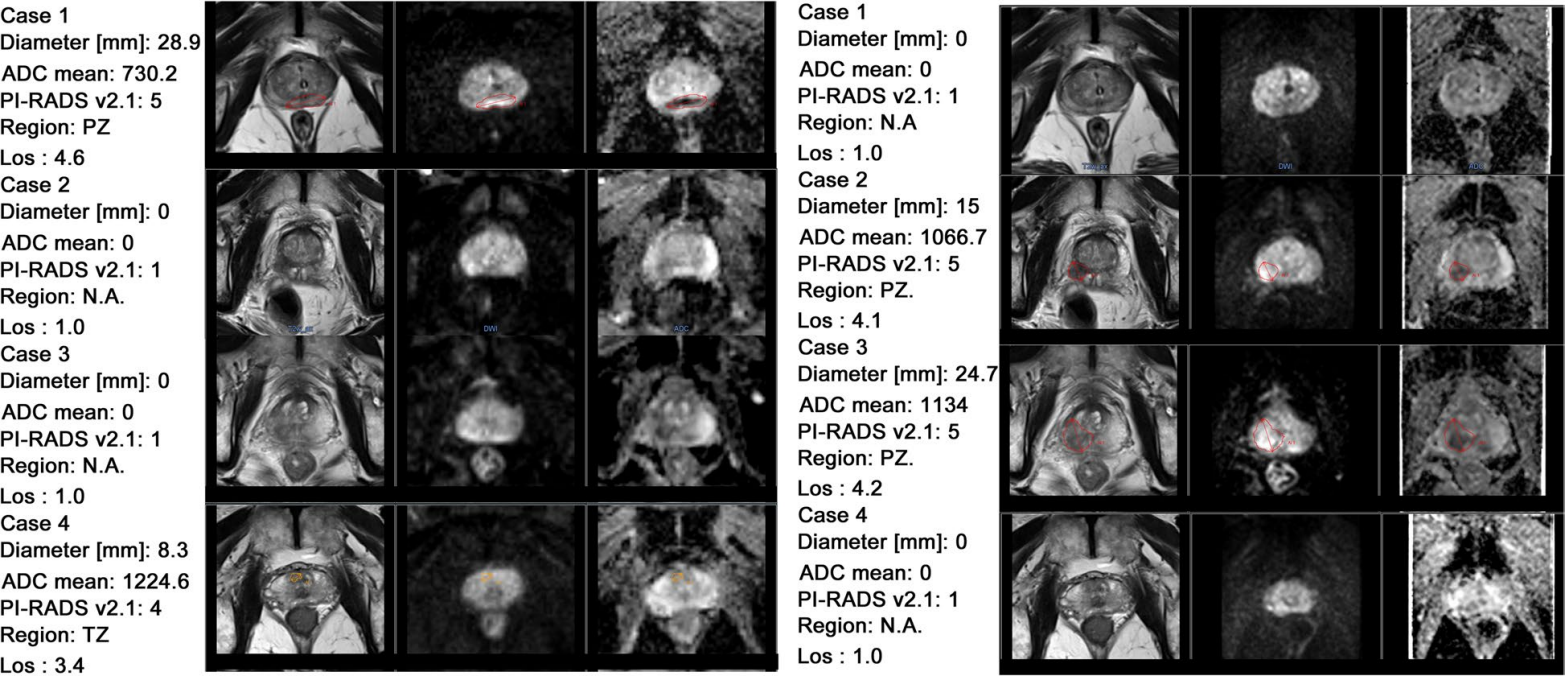
Examples of DL-CAD diagnosis using f-DWI (**a**) and z-DWI (**b**). The red or orange ROIs indicate highly suspected PCa lesions detected by the system. Cases without ROIs indicate no suspected PCa lesion was detected. Case 1. A 77-year-old man with an inflammatory lesion in the central zone (CZ). DL-CAD using f-DWI detected a fake cancer lesion in PZ, which was a rectal artifact. Case 2. A 62-year-old man with a PCa lesion (Gs = 3 + 4) in the right PZ, which was missed by DL-CAD with the use of f-DWI possibly due to rectal distortion and artifacts. Case 3. A 71-year-old man with an inflammatory lesion in the right PZ, which was detected by DL-CAD using z-DWI. Case 4. A 73-year-old man with a PCa lesion (Gs = 4 + 3) in the transition zone (TZ) which was missed by DL-CAD using z-DWI

Examples of DL-CAD diagnosis using f-DWI and z-DWI are shown in Fig. 5. Based on subjective visual evaluation, prostate deformation, artifacts, and lesion signal intensity on DWI and ADC values are possible reasons resulting in DL-CAD misdiagnosis.

**Fig. 6:**
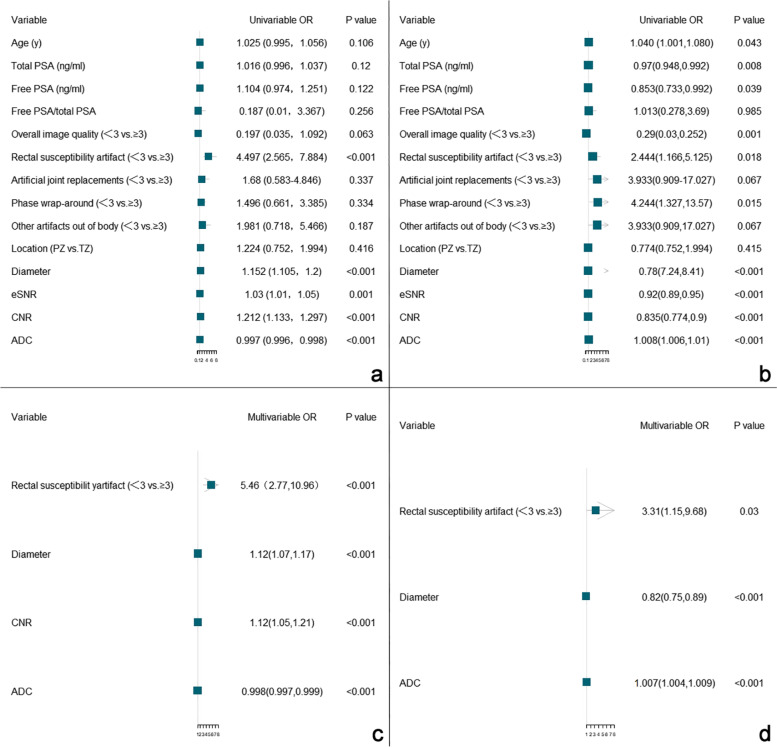
Logistic regression analyses showing variables associated with wrong detections. **a** and **b** is the univariable logistic regression analysis of benign and malignant lesions, respectively. **c** and **d** is the multivariable logistic regression analyses of benign and malignant lesions, respectively. PSA, prostate-specific antigen; PZ, peripheral zone; TZ, transition zone; eSNR, estimated signal-to-noise ratio

As shown in Fig. 6, CNR was an independent risk factor for false positives of DL-CAD (odds ratio [OR], 1.12; 95% CI, 1.05-1.21; *P* < .001) and rectal susceptibility artifacts, diameter, and ADC were independent risk factors associated with both false positive detections (OR_rectal susceptibility artifact,_ 5.46; 95% CI, 2.77-10.96; OR_diameter_, 1.12; 95% CI, 1.07-1.17; OR_ADC,_ 0.998; 95% CI, 0.997-0.999; all *P* < .001) and false negative detections (OR_rectal susceptibility artifacts_, 3.31; 95% CI, 1.15-9.68; OR_diameter_, 0.82; 95% CI, 0.75-0.89; OR_ADC_, 1.007; 95% CI, 1.004-1.009; all *P* ≤ .03) of DL-CAD.

## Discussion

Our study has two main contributions. First, we compared PCa detection performance of the DL-CAD system with the use of z-DWI and f-DWI, finding that the DL-CAD system exhibited significantly better PCa detection performance based on z-DWI than using f-DWI. It indicates that z-DWI may be a way towards more consistent and better image quality. Second, risk factors that affected the diagnostic performance of the DL-CAD system in the assessment of PCa were identified. As these types of image artifacts are common in prostate MRI, the risk factors that interfere with one DL-CAD system may have similar effects in other DL-CAD systems with different network structures and parameter settings. Understanding these risk factors might be helpful for standardizing prostate MRI scanning guidelines for DL-CAD analysis, customizing the corresponding DL-CAD training strategies, and improving the diagnostic accuracy and generalization of DL-CAD.

DWI acquired by zoomed-FOV technology has provided better image quality than that obtained with other technologies [10, 15, 18, 32, 34]. However, due in part to the physiological limitations of visually identifying subtle differences among lesions, the improvement in image quality did not significantly improve the subjective evaluation performance of radiologists for PCa detection in many previous studies [15, 34]. Given the ability of DL-CAD to mine the sub-pixel level, previous radiomic study found that a radiomics model based on z-DWI had a higher diagnostic accuracy, sensitivity, and specificity than a model based on f-DWI [17]. Partly differing from previous result, the improvement of PCa detection performance of the DL-CAD using z-DWI primarily comes from the improvement in sensitivity. We found that the sensitivity of DL-CAD using z-DWI for detecting lesions improved by 20%. However, its specificity for detecting lesions was reduced by 15%. Our results indicate that the observed operating point of DL-CAD using z-DWI was shifted in favor of higher sensitivity, but considering the superior ROC curves, z-DWI achieved superior specificity at given sensitivity levels.

In contrast to the radiomic model which was constructed with explicable texture feature information, the training and diagnosis process of DL-CAD is much more complex. We used common clinical research methods to find risk factors contributing to diagnostic errors from the macroscopic level of DL-CAD. We found that CNR was positively associated with false positives of DL-CAD, whilst ADC was negatively associated with false positives of DL-CAD. It indicates that parameter settings producing on average lower ADC values and higher CNR in PCa lesions in DWI might be helpful to improve the performance of DL-CAD using DWI sets.

Because the lesions in z-DWI have lower ADC values but higher CNR in both benign and malignant lesions than those in f-DWI, it is not surprising that DL-CAD using z-DWI detected more true positives but had increased false positives. Therefore, strategies to overcome these problems will need to be determined before applying z-DWI to existing f-DWI based DL-CAD systems, e.g., by further training the DL-CAD with z-DWI data in the future.

Consistent with our clinical observations, we found that the severity of rectal susceptibility artifacts is an independent high-risk factor for both false positives and false negatives of DL-CAD. Severe artifacts in the rectal region led to signal gain or loss in adjacent prostate tissue and to local gland deformation of the prostate, which impairs the performance of DL-CAD which relies on an accurate co-registration of T2WI and DWI. Therefore, we hold that the reduction of rectal susceptibility artifacts was one of the main reasons why the DL-CAD diagnostic accuracy using z-DWI was improved. It is also indicated that good bowel preparation before prostate MRI examination may help to maintain the accuracy and stability of DL-CAD.

Another interesting finding of our study which is inconsistent with our initial expectations is that noise and phase wrap-around were not found to be independent risk factors that interfered with the diagnostic efficiency of DL-CAD. These aspects may not be important considerations for developing improved DWI scanning strategies of DL-CAD for PCa diagnosis. According to the results, artifacts caused by artificial joint replacements and other artifacts out-of-body in DWI also were not key factors resulting in the misdiagnoses of DL-CAD. However, considering few patients suffering these artifacts, this result still needs to be further verified by larger samples.

Our study had limitations. First, only one trained DL-CAD based on full-FOV DWI was used for the comparison of DWI sets. Although the effects of reduced image quality are typically not limited to a single model, whether the risk factors we have identified affect the performance of other models still needs further verification. Second, only DWI images obtained from a single manufacturer were included for comparison. Results from multi-vendor datasets obtained from multiple imaging centers are needed to verify our results. Third, because in our clinic routine, active surveillance instead of biopsy is typically selected for patients with elevated PSA but negative MRIs, it is hard for us to evaluate the performances of DL-CAD on PCa patients with negative MRIs. Therefore, only patients with prostate lesions with definite boundaries on all MR images were included, there might be a potential source of selection bias. Fourth, a reduced FOV may prevent the visualization of lymph nodes, a full-FOV DWI is still needed to study lymph nodes. Finally, in our study, targeted biopsies were used as reference standards. Whole-mount histopathology may have improved the accuracy of the agreement between the MR images and the histopathology.

## Conclusions

In conclusion, z-DWI has the potential to improve the detection performance of a prostate MRI based DL-CAD system.

## Data Availability

The datasets used and/or analyzed during the current study are available from the corresponding author on reasonable request.

## Abbreviations

DL-CAD: Deep learning-based computer aided diagnosis
PCa: Prostate cancer
ssEPI: Single-shot echo-planar imaging
f-DWI: Full-field-of-view DWI
z-DWI: Zoomed-field-of-view echo-planar DWI
AUC: Area under the curve
CNR: Contrast-to-noise ratio
OR: Odds ratio
ADC: Apparent diffusion coefficient
FOV: Field-of-view
PI-RADS: Prostate Imaging Reporting and Data System
GGG: Gleason Grade Group
LoS: Case-based level of suspicion
ROI: Region-of-interest
FROC: Free-response receiver operating characteristics
VIF: Variance inflation factor
GS: Gleason score
SD: Standard deviation
eSNR: Estimated signal-to-noise ratio
